# NLRP3 Inflammasome Promotes the Progression of Acute Myeloid Leukemia *via* IL-1β Pathway

**DOI:** 10.3389/fimmu.2021.661939

**Published:** 2021-06-15

**Authors:** Chaoqin Zhong, Ruiqing Wang, Mingqiang Hua, Chen Zhang, Fengjiao Han, Miao Xu, Xinyu Yang, Guosheng Li, Xiang Hu, Tao Sun, Chunyan Ji, Daoxin Ma

**Affiliations:** ^1^ Department of Hematology, Qilu Hospital of Shandong University, Jinan, China; ^2^ Department of Hematology, Shandong Yantai Mountain Hospital, Yantai, China; ^3^ Department of Emergency, Qilu Hospital of Shandong University, Jinan, China

**Keywords:** inflammasome, NLRP3, IL-1β, acute myeloid leukemia, progression

## Abstract

NLRP3 inflammasome has been reported to be associated with the pathogenesis of multiple solid tumors. However, the role of NLRP3 inflammasome in acute myeloid leukemia (AML) remains unclear. We showed that NLRP3 inflammasome is over-expressed and highly activated in AML bone marrow leukemia cells, which is correlated with poor prognosis. The activation of NLRP3 inflammasome in AML cells promotes leukemia cells proliferation, inhibits apoptosis and increases resistance to chemotherapy, while inactivation of NLRP3 by caspase-1 or NF-κB inhibitor shows leukemia-suppressing effects. Bayesian networks analysis and cell co-culture tests further suggest that NLRP3 inflammasome acts through IL-1β but not IL-18 in AML. Knocking down endogenous IL-1β or anti-IL-1β antibody inhibits leukemia cells whereas IL-1β cytokine enhances leukemia proliferation. In AML murine model, up-regulation of NLRP3 increases the leukemia burden in bone marrow, spleen and liver, and shortens the survival time; furthermore, knocking out NLRP3 inhibits leukemia progression. Collectively, all these evidences demonstrate that NLRP3 inflammasome promotes AML progression in an IL-1β dependent manner, and targeting NLRP3 inflammasome may provide a novel therapeutic option for AML.

## Highlights

- NLRP3 inflammasome is over-expressed and highly activated in AML bone marrow, and correlates with poor prognosis in AML.
- NLRP3 inflammasome promotes AML progression in an IL-1β dependent manner.

## Introduction

Acute myeloid leukemia (AML) is a hematopoietic stem cell malignancy characterized by ineffective hematopoiesis and excessive proliferation of immature myeloid cells (1). With the enormous cytogenetic and molecular heterogeneity of AML identified, targeted therapy has made great progresses; however, the overall survival of AML patients remains unsatisfied, highlighting the urgent need for novel therapeutic strategies. Abnormalities of immune system have been recognized in AML, and our previous studies have demonstrated that imbalanced T-helper (Th) cells contribute to AML pathogenesis (2, 3). Nevertheless, the detailed immunological mechanism of AML remains unclear.

Inflammasomes are cytosolic multi-protein complexes involved in innate immune response. NLRP3 inflammasome containing NLRP3, ASC, and pro-caspase-1 is one of the best-characterized inflammasome (4, 5) and has been proven to be involved in AML in our previous study (3). The exact mechanism of NLRP3 inflammasome activation is still under investigation, and a spatially and temporally separated two-signal process including priming and activation has been generally accepted (6). Signal one up-regulates transcription of pro-IL-1β and NLRP3 *via* NF-κB activation by sensing PAMPs/DAMPs in a TLRs-, NLRs-, TNFR1- and IL-1R1-dependent manner, and signal two is the assembly of the inflammasome complex and activation of caspase-1 (4, 7). When stimulated, the NLRP3 inflammasome formation results in caspase-1 activation and subsequently converts pro-inflammatory cytokine pro-IL-1β or pro-IL-18 into its mature bioactive form. Mature IL-1β or IL-18 is then released outside the cell (4, 7). Lipopolysaccharide (LPS) can trigger the NLRP3 inflammasome activation in human cells *via* TLR4 pathway (8). In addition, IL-1β itself induces synthesis of pro-IL-1β through IL-1RI-MyD88-NF-κB pathway, resulting in a positive feedback loop (9).

Though, the controversial role of NLRP3 inflammasome in the carcinogenesis has been debated (10–12), increasing evidences emphasize that NLRP3 inflammasome promotes chronic inflammatory response which contributes to cancer initiation, development and progression (13–16). NLRP3 inflammasome effector cytokine IL-1β or IL-18 is also proved to be involved in cell proliferation, differentiation and considered to play a pivotal role in tumorigenesis (9, 17–21). It was reported that high concentrations of serum IL-1β and IL-18 are strictly correlated to poor prognosis of cancer patients (10, 12). Moreover, multiple studies showed that inflammasomes and IL-1 signaling manifested carcinogenic roles in head and neck squamous cell carcinoma, gastric carcinoma, breast cancer, lung cancer, and other cancers (9, 13, 16, 22–26). Therefore, antagonists, such as small molecules or antibodies targeting components of the inflammasome complex are developed (9, 27). Our previous studies also demonstrated that the polymorphisms of NLRP3 inflammasome might be involved in the pathogenesis of hematological malignancies, such as lymphoma (28), multiple myeloma (29), myelodysplastic syndromes (30) and AML (31). Moreover, we found that aberrant NLRP3 expression associated with Aryl hydrocarbon receptor (AHR) may contribute to Th cells imbalance in AML patients (3). However, the detailed role of NLRP3 inflammasome in the progression of AML remains to be investigated.

Here, we showed for the first time that highly activated NLRP3 inflammasome in AML cells plays carcinogenetic roles in an IL-1β dependent manner. Furthermore, inhibition of NLRP3 inflammasome activation manifests anti-leukemia effects both *in vitro* and *in vivo*. In conclusion, our results identify that activated NLRP3 is a critical biological actor in the pathogenesis of AML and suggest novel strategies for therapeutic intervention.

## Materials and Methods

### Patients and Specimens

Bone marrow aspirates included in this study were from 84 newly diagnosed (ND) AML patients and 16 age- or gender-matched controls. Because bone marrow aspiration is an invasive procedure, healthy donors and individuals with mild iron deficiency anaemia without immunological changes were used as controls. These specimens were collected at Qilu Hospital of Shandong University. Bone marrow mononuclear cells (BM-MNCs) were isolated by density-gradient centrifugation with Ficoll-Hypaque (Haoyang biotechnology company, Tianjin, China). If red cell contamination from BM-MNCs was more than thirty percent, red blood cell lysis was performed. A total of 2×10^6^ BM-MNCs were stabilized in TRIZOL and stored at -80°C for RNA extraction. BM-MNCs obtained from ND AML patients with high marrow blast counts (20 patients with >70%, 8 patients with 60~70% BM blasts, respectively) were used as primary leukemia cells immediately for cell culture experiment. All studies were performed with the patients’ written informed consent at the beginning of the trial and were approved by the Medical Ethical Committee of Qilu Hospital of Shandong University (KYLL-2016-300). The related clinical characteristics for AML patients in our study are presented in **Table 1**.

**Table 1:** Clinical characteristics of ND AML patients and controls.

Features	ND AML (n=84)	Control (n=16)
Gender (male/female)	40/44	6/10
Age (years, median, range)	45.5 (12,83)	49 (27,76)
WBC (×10^9^/L)	20.5 (1.21,356.87)	5.08±1.71
Neu%	22.1 (0,94.1)	70.07±8.22
Mon%	29.5 (0,84.6)	0.23±0.06
RBC (×10^12^/L)	2.18 (1.41,5.07)	3.22±0.74
HGB (g/L)	70.5 (44,149)	94.31±30.52
PLT (×10^9^/L)	38 (8,310)	187.92±100.98
BM leukemic blasts (%)	86(0.71,97)	
FAB subtypes		
M2	2	
M3	14	
M4	12	
M5	46	
Unclassified	10	

ND, newly diagnosed; WBC, white blood cells; RBC, red blood cells; HGB, haemoglobin; PLT, blood platelet; BM, bone marrow.

### Cells and Reagents

THP-1 and U937 cells were purchased from Shanghai Institutes for Biological Sciences of China, and murine AML cell line C1498 was obtained from ATCC (Cat. TIB-49). THP-1, U937 or primary leukemia cells were cultured in RPMI 1640 culture medium (Gibco, USA) supplemented with 10% fetal bovine serum (FBS, Gibco, USA) and 1% penicillin/streptomycin (Gibco, USA). C1498 cells were cultured in DMEM medium containing 10% FBS and 1% penicillin/streptomycin. And cells were incubated at 37°C in humidified atmosphere with 5% CO_2._ The NLRP3 lentiviral over expression system was established by inserting the cDNA of NLRP3 in the open reading frame of the vector Ubi-MCS-3FLAG-SV40-EGFP-IRES-puromycin (GeneChem Company, Shanghai, China) using Age I/Nhe I digestion. Recombinant human IL-1β, IL-18, anti-IL-1β and anti-IL-18 were purchased from R&D Systems (Minneapolis, MN, USA). LPS and ATP were purchased from Sigma-Aldrich. The caspase-1 inhibitor Z-YVAD-FMK was purchased from Alexis Biochemicals (Heidelberg, Germany). The NF-κB inhibitor Bay11-7082 was purchased from Selleck chemicals (Selleckchem, USA). Dimethylsulfoxide (DMSO) was purchased from Sigma (USA). The chemotherapy drugs we used are nucleic acid synthesis inhibitors [Aadriamycin (ADR) and Daunorubicin (DNR)] and cell cycle inhibitor [Cytosine Arabinoside (Ara-C)]. They were dissolved in normal saline and diluted in RPMI 1640 medium immediately before use. Primary antibodies against NLRP3 (#15101), NF-κB p65(# 8242), phospho-NF-κB p65 (NF-κB pp65) (#3033), caspase-1 (#3866), pro-IL-1β (#12703), cleaved IL-1β (#83186), Bcl-2 (#3498), C-myc (#5605), and cleaved PARP (#9548) used for Western blot were obtained from Cell Signaling Technology (Beverly, MA, USA); and antibodies against caspase-9 (ab202068), β-tubulin (ab0039), cleaved caspase-3 (ab13847), BAX (ab199677) and β-actin (ab227387) were purchased from Abcam (Cambridge, UK). The secondary antibodies were obtained from Millipore (USA). All reagents were dissolved and preserved following the manual’s instructions.

### NLRP3 Activation and Inactivation in Leukemia Cells

NLRP3 inflammasome can be activated by LPS or LPS+ATP (8). Therefore, in our study, NLRP3 activation for THP-1 or primary leukemia cells was conducted with LPS (1μg/mL) or followed by ATP (5 mmol/L). NLRP3 effector cytokine IL-1β (10 ng/mL) or IL-18 (10 ng/mL) was also added into leukemia cells to investigate the role of NLRP3 inflammasome in AML. NLRP3 inactivation was performed by inhibiting caspase-1 or NF-κB pathway. To inhibit caspase-1, leukemia cells were pretreated with caspase-1 inhibitor Z-YVAD-FMK at 10 umol/L for 1 hour. For the inhibition of NLRP3 inflammasome activation *via* NF-κB signaling pathway, NF-κB inhibitor Bay11-7082 (10 umol/L) was used to treat leukemia cells for 1hour. Moreover, ADR (200 μg/L) or DNR (20 μg/L) was added into leukemia cells to determine the role of NLRP3 inflammasome in antitumor activity of chemotherapy drug. After being incubated for 24, 48, 72 or 96 hours, cells were collected for cell proliferation and apoptosis analysis. In addition, cells were harvested for qRT-PCR and Western blot analysis and the supernatants were collected for ELISA analysis.

### Cell Co-Culture Experiments

THP-1 cells were stimulated with LPS (1μg/mL) for 6 hours followed by ATP (5mmol/L) for 1 hour to activate NLRP3 inflammasome. After being washed with normal saline for three times, a total of 100uL NLRP3-activated THP-1 cells (5 × 10^4^ cells/mL) in the upper chamber were co-cultured with 600µL primary leukemia cells (1 × 10^6^ cells/mL) in the bottom chamber using Transwell chamber (24-well, 0.4 µm, Corning, NY, USA). Anti-IL-1β or anti-IL-18 was added for neutralization. After being incubated for 48 hours, primary leukemia cells in the bottom chamber were harvested for apoptosis assay.

### siRNA Knockdown of IL-1β

THP-1 cells were plated into 24-well plates (2.5× 10^5^ cells/well) and transfected with IL-1β siRNA (2.0 µmol/L) using exfect 2000 (VAZAME, China) for 8 hours according to manufacturer’s protocol. The IL-1β siRNA (Genepharma, China) was as below: sense 5’ GGU GAU GUC UGG UCC AUA UTT 3’, antisense 5’ AUA UGG ACC AGA CAU CAC CTT 3’, while the negative control sequence was: sense 5’ UUC UCC GAA CGU GUC ACG UTT 3’, antisense 5’ ACG UGA CAC GUU CGG AGAATT 3’. The IL-1β expression, cell proliferation and drug sensitivity were determined 48 hours later.

### NLRP3 Lentiviral Infection of C1498

C1498 cells were infected with NLRP3-GFP or Ctrl-GFP lentivirus (Ubi-MCS-3FLAG-SV40-EGFP-IRES-puromycin). Puromycin was used to gain the stable infection cells and fluorescence microscope was used to determine the infection efficiency after 48 hours. The expression of NLRP3 was analyzed at both the mRNA and protein levels to verify the efficiency of infection. Then the lentivirus infected C1498 cells were stimulated with LPS (1μg/mL) to activate NLRP3 inflammasome and the expressions of NLRP3 components were examined by Western blot and qRT-PCR.

### AML Murine Models

#### NLRP3-Upregulated AML Murine Model

Female WT C57BL/6J mice were obtained from the Laboratory Animal Center, Shandong University. C1498 cells were intravenously injected into mice to establish the AML model. The experimental mice were injected with 1 × 10^6^ NLRP3-GFP C1498 cells, and mice injected with Ctrl-GFP C1498 cells were used as controls, and normal mice were injected with PBS. AML mice were sacrificed 21 days later. The complications (spleen, the liver and bone barrow invasion) were analyzed and the expressions of NLRP3 related molecules were determined. The survival was also observed for another cohort of AML mice.

#### NLRP3-Knockout AML Murine Model

*NLRP3* ^-/-^ mice of C57BL/6J background were kindly provided by Dr. Rongbin Zhou (Chinese University of Science and Technology). The retrovirus vector with intracellular domain of MLL-AF9 (MSCV-MLL-AF9-IRES-GFP) was kindly provided by Dr. Hui Cheng (Institute of Hematology, Chinese Academy of Medical Sciences). C-kit^+^ cells from *NLRP3^-/-^* mice transfected with MSCV-MLL-AF9-IRES-GFP (10^7^ cells/host) and BM-MNCs from C57BL/6J mice (10^7^ cells/host) were transplanted into lethally irradiated C57BL/6J recipients. In control group, recipients were transplanted with 10^7^ C-kit^+^ cells from WT C57BL/6J mice transfected with MSCV-MLL-AF9-IRES-GFP and 10^7^ BM-MNCs from C57BL/6J mice. BM-MNCs from C57BL/6J mice were used as protective cells for lethally irradiated C57BL/6J recipients in experimental and control group. On day 30 after transplantation, *NLRP3^-/-^* AML mice were verified by using peripheral blood from the lateral tail vein with FACS Aria II sorter (BD Biosciences).

All mice were six to eight week old and were maintained in a specific pathogen-free environment. Animal protocols were approved by the Animal Ethics committee of Qilu Hospital, Shandong University.

### Cell Proliferation Assays

After different treatments, the cells were incubated with 10uL CCK8 (Beyotime, China) for 3 hours. The absorbance was measured at 450 nm. Each sample was conducted in triplicate.

### Apoptosis Assays

Apoptosis was performed with the Annexin V/PI apoptosis detection kit (BestBio, Shanghai, China) according to the manufacturer’s protocol. Cells were harvested after different treatments and washed twice with PBS. Then cells were re-suspended in 400 μL binding buffer, and stained with 5μL Annexin V for 15 minutes, 10μL PI for another 5 minutes in the dark at 4°C. The percentages of apoptotic cells (Annexin V+/PI- as early apoptosis, and Annexin V+/PI+ as late apoptosis) were analyzed immediately by Galios flow cytometry (Beckman Coulter, CA, USA).

### Quantitative Reverse Transcriptase PCR (qRT-PCR)

Total RNA was extracted from AML primary leukemia cells or THP-1 U937 and C1498 cell line cells using TRIZOL (Invitrogen, USA). The total RNA concentration and purity were quantified by spectrophotometer (Eppendorf, GER). Reverse transcription was performed at 37°C for 15 minutes followed by 85°C for 10 seconds using Prime Script RT reagent Kit Perfect Real Time (Takara Bio Inc, Japan). Quantitative PCR was operated in duplicate on Light Cycler 480II real-time PCR system (Roche, Switzerland) with SYBR Green Real-time PCR Master Mix kit (Toyobo, Japan). The PCR contained 3.2 μL ddH2O, 5 μL of 2×SYBR Green Real-time PCR Master Mix, 0.4 μL of the forward and reverse primers, and 1 μL of cDNA in a final volume of 10 μL. PCR conditions were performed as follows: 95°C for 10 minutes followed by 40 cycles (95°C for 20 seconds and 60°C for 1 minutes). The primer sequences for the relevant genes were shown in **Table 2**. To determine the specificity of the PCR reaction, melting curves were routinely analyzed. All experiments were conducted according to the manual’s instructions. Relative gene expression was expressed relative to endogenous control GAPDH and calculated using 2 ^–ΔCT^ method.

**Table 2:** The sequences of PCR primers.

gene	Primers (5’-3’)	
hNLRP3	CAGACTTCTGTGTGTGGGACTGA	TCCTGACAACATGCTGATGTGA
hcaspase-1	AAATCTCACTGCTTCGGACATG	GGAACTTGCTGTCAGAGGTCTT
hASC	TGGATGCTCTGTACGGGAAG	CCAGGCTGGTGTGAAACTGAA
hIL-1β	ATGATGGCTTATTACAGTGGCAA	GTCGGAGATTCGTAGCTGGA
hIL-18	GCTTGAATCTAAATTATCAGTC	GAAGATTCAAATTGCATCTTAT
hNF-κB	CTTCCAAGAAGAGCAGCGTG	TTTCGGTTCACTCGGCAGAT
hIL-1R	TGCCTTTCCACCTGCTTTCT	TCAAGACGTGACATCCCTGC
hGAPDH	GCTCTCTGCTCCTCCTGTTC	GTTGACTCCGACCTTCACCT
mNLRP3	CCGAGAAGGCTGTATCCC	CGTGTCATTCCACTCTGGCT
mIL-1β	TGCCTITGACAGTGATG	TGATGTGCTGCTGCGAGATT
mNF-κB	GACATGGTGGTTGGCTTTGC	CCTCCGCCTTCTGCTTGTAG
mcaspase-1	CGTACACGTCTTGCCTCAT	CTTCACCATCTCCAGAGC
mGAPDH	GGACACTGAGCAAGAGAGGC	TTATGGGGGTCTGGGATGGA

### Enzyme-Linked Immunosorbent Assay (ELISA)

The BM plasma samples were collected from EDTA stabilized bone marrow of 70 ND AML patients and 15 controls, and supernatants of primary leukemia cells or THP-1 cells were collected and stored at -80°C for determination of cytokines. Human IL-1β ELISA kit was purchased from R&D Systems (Minneapolis, MN, USA) and IL-18 ELISA kit was purchased from eBioscience (San Diego, CA). ELISA was performed in accordance with the manufacturer’s instructions.

### Western Blot Analysis

Cells were collected, washed twice with PBS, and then lysed with RIPA buffer (Beyotime, China) containing protease inhibitor compound (Beyotime, China) on ice. Bicinchoninic acid (BCA) protein assay Kit (Beyotime, China) was applied to measure the concentration of proteins. Proteins extracts (30 μg) were loaded onto 10% SDS-PAGE and then electro-transferred onto nitrocellulose membranes (Millipore, Bedford, Massachusetts, USA). After being blocked with 5% nonfat milk for 1 hour at room temperature, the membranes were incubated overnight with specific primary antibodies at 4°C followed by being incubated with HRP-conjugated secondary antibodies at room temperature for 1 hour. Protein bands were detected by FluorChem E Chemiluminescent imaging system (ProteinSimple, San Jose, CA, USA) after washing.

### Statistical Analysis

GraphPad Prism 5.0 was applied in diagrams drawing. Statistical analysis was conducted with raw data using SPSS 20.0 software. Shapiro-Wilk test was used for normality tests. Data normally distributed were analyzed by Student’s t-test or paired t tests. Otherwise, comparisons between groups were performed using Mann-Whitney U test for non-paired data and Wilcoxon signed rank test for paired data. Data in results are expressed as the mean ± s.e.m, except where stated otherwise. To represent the complicated correlation structure among the genes in NLRP3 inflammasome, score-based hill-climbing greedy search algorithm using mRNA expression data was applied to learn the Bayesian network (32). * *P* <.05, ** *P* <.01 and *** *P*<.0001 were considered statistically significant.

## Results

### NLRP3 Inflammasome Is Over-Expressed and Highly Activated in AML, Which Plays Leukemia-Promoting Effects *In Vitro*

**Figure 1:**
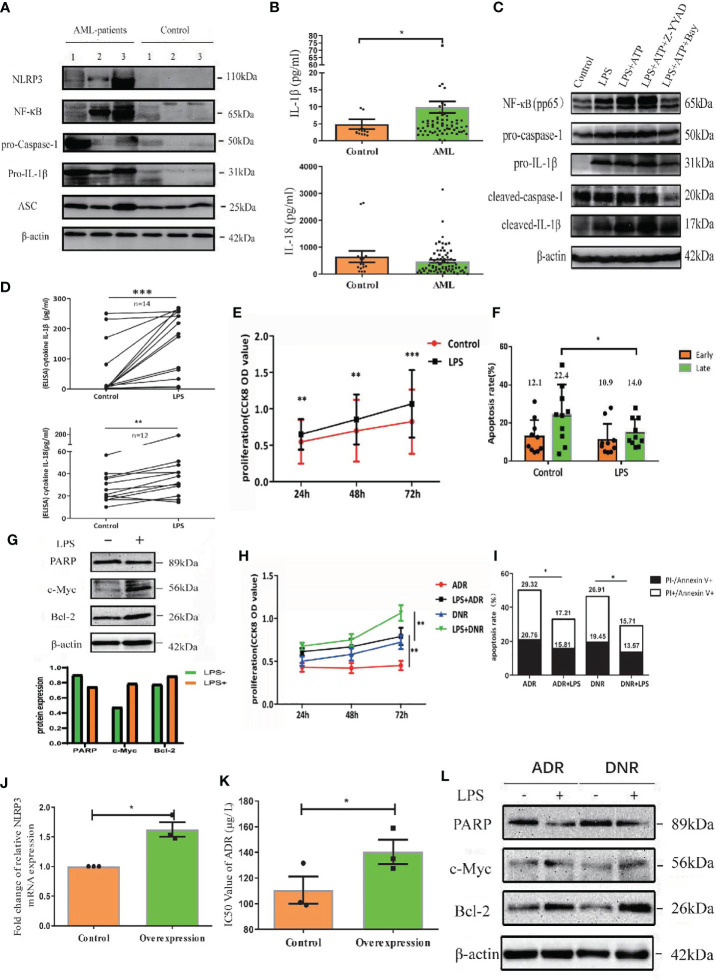
NLRP3 inflammasome is over-expressed and highly activated in AML, which plays leukemia-promoting effects *in vitro*. **(A)** The western blot results of NLRP3, NF-κB, caspase-1, pro-IL-1β and ASC in BM-MNCs from ND AML patients (n=3) and controls (n=3). **(B)** The concentrations of IL-1β and IL-18 in BM supernatant from ND AML patients (n=70) in comparison to controls (n=15). **(C)** The Western blot results of pNF-κB, pro-caspase-1, pro-IL-1β, cleaved caspase-1 and cleaved IL-1β were showed in primary leukemia cells after different treatment. β-actin is used as a loading control. **(D)** LPS stimulated the secretion of IL-1β and IL-18 into the supernatants from cultured leukemia cells. **(E)** CCK8 analysis was performed to detect the proliferation of leukemia cells 24, 48 and 72 hours after LPS stimulation (n=10). **(F)** The quantified apoptosis rate was shown (n=10). **(G)** The western blot results of PARP, C-myc and Bcl-2 in primary leukemia cells after LPS stimulation. **(H)** CCK8 analysis for the proliferation of primary leukemia cells with or without LPS 24, 48 and 72 hours after treatment with ADR, DNR (n=10). **(I)** The apoptosis results of primary leukemia cells with or without LPS after treatment with ADR, DNR (n=6). **(J)** The expression of NLRP3 in U937 cell line was enhanced after being transfected with lentivirus (n=3). **(K)** The IC50 value of ADR for U937 cells was higher after upregulating NLRP3 expression (n=3). **(L)** PARP, C-myc and Bcl-2 in primary leukemia cells after LPS stimulation (n=1). β-actin is used as a loading control. *P < 0.05; **P < 0.01; ***P < 0.001.

To determine the expression of NLRP3 inflammasome in AML patients, we examined NLRP3 inflammasome associated molecules in BM-MNCs isolated from 63 newly diagnosed AML patients by qRT-PCR. The inflammasome transcripts from AML samples were significantly up-regulated compared with those from controls, including NLRP3, IL-1β, NF-κB and IL-1R, whereas the expression of ASC, caspase-1 or IL-18 showed no significant difference between two groups (**Supplementary Figure 1A**). The protein level of NLRP3 inflammasome was further quantified by western blot, in which NLRP3, NF-κB, pro-caspase-1 and pro-IL-1β were dramatically increased in BM-MNCs of ND AML patients, while they were hardly detected in BM-MNCs of controls (**Figure 1A**, **Supplementary Figure 1E**). Furthermore, as the activation of NLRP3 inflammasome is associated with the secretion of cytokines IL-1β and IL-18 (7), we measured the concentrations of IL-1β and IL-18 in the supernatants of BM from 70 ND AML patients and 15 controls using ELISA. The results confirmed that the concentration of IL-1β was significantly elevated in ND AML patients compared with controls, whereas the level of IL-18 was similar to controls (**Figure 1B**).

The effect of NLRP3 activation in AML has not been evaluated. To clarify this effect, we firstly activated the NLRP3 inflammasome using LPS in primary AML leukemia cells as described previously (8). The result showed that LPS significantly increased the transcription levels of caspase-1 and IL-1β (**Supplementary Figure 1B**). Moreover, LPS upregulated the protein expression of pNF-κB, pro-caspase-1, pro-IL-1β, cleaved caspase-1 or cleaved IL-1β (**Figure 1C**, left two columns, **Supplementary Figure 1F**), and promoted the secretion of IL-1β or IL-18 into culture medium (**Figure 1D**). After verifying the successful activation of NLRP3 inflammasome, CCK8 was used to determine the cell proliferation and flow cytometry analysis was performed to analyze apoptosis. Our results demonstrated that NLRP3 activation significantly promoted the cell proliferation and decreased the cell apoptosis of AML cells (**Figures 1E, F**, **Supplementary Figure 1C**). After determining the proliferation related protein by western blot, we found LPS stimulation upregulated the expression of onco-protein Bcl-2 or C-myc (**Figure 1G**). Furthermore, we explored the role of NLRP3 inflammasome activation in AML chemotherapy, and found that the killing effect of ADR or DNR on leukemia cells was reversed by LPS-induced NLRP3 activation (**Figures 1H, I**, **Supplementary Figure 1D**). Moreover, we upregulated NLRP3 expression by lentivirus transfection in leukemia cells to study its effect on drug resistance (**Figure 1J**). The result showed that NLRP3 overexpression increased the IC50 of ADR in U937 leukemia cells (from 114.03 to 143.06 µg/L), indicating the response to ADR was inhibited by increased NLRP3 expression and activation (**Figure 1K**). Mechanistically, NLRP3 activation significantly inhibited the drug-induced expression of PARP-1, and promoted C-myc and Bcl-2 expression (**Figure 1L**, **Supplementary Figure 1G**). These results indicated that NLRP3 inflammasome activation plays a leukemia-promoting role in AML.

### Up-Regulation of NLRP3 Promotes AML Progression and Shortens Survival in AML Mice

To further investigate the role of NLRP3 inflammasome in AML *in vivo*, we up-regulated NLRP3 expression in AML murine cell line C1498 and explored their progression in mice. After successful transfection of C1498 cells with NLRP3-GFP or Ctrl-GFP lentivirus (**Supplementary Figure 2A**), we found that NLRP3-GFP lentivirus significantly up-regulated NLRP3 expression at both mRNA and protein levels (**Supplementary Figures 2B, C**). Moreover, after LPS stimulation, the mRNA or protein level of IL-1β was much higher in NLRP3-GFP transfected C1498 cells than Ctrl-GFP control (**Supplementary Figures 2D, E**).

**Figure 2:**
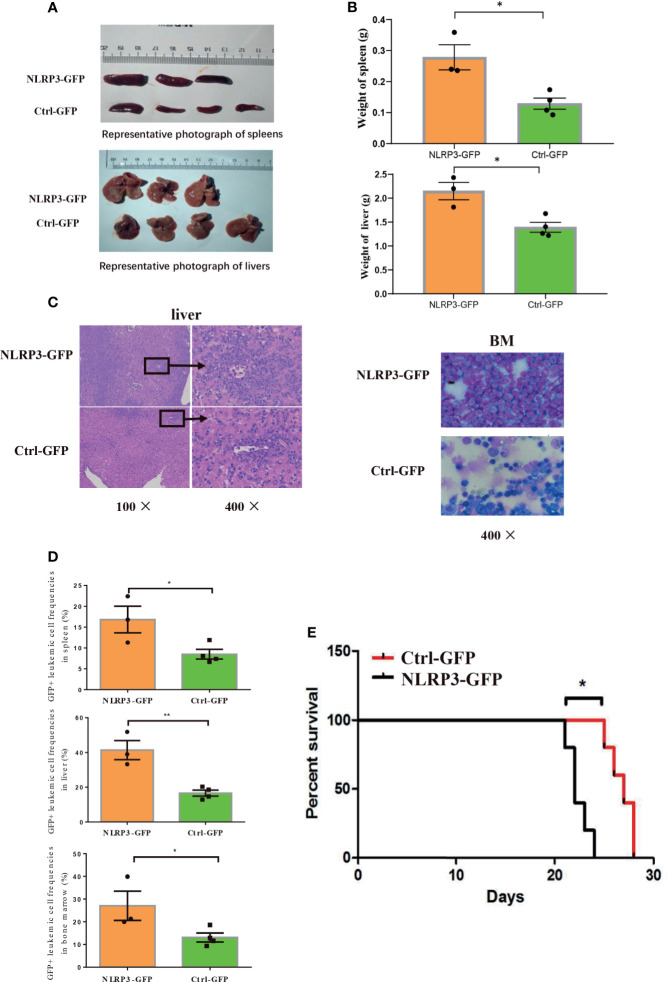
Up-regulation of NLRP3 promotes AML progression and shortens survival in AML mice. **(A)** Representative photographs of spleen and liver from NLRP3-GFP mice (n=3), Ctrl-GFP mice (n=4) are compared, **(B)** with the analysis of the weight of spleens and livers of NLRP3-GFP (n=3) and Ctrl-GFP mice (n=4). **(C)** Hematoxylin and eosin–stained histopathology sections of a representative liver and bone marrow from Ctrl-GFP and NLRP3-GFP mice (100× or 400×). **(D)** The leukemia cells (GFP+ cells) in spleen, liver and bone marrow from NLRP3-GFP (n=3) and Ctrl-GFP (n=4) mice. **(E)** Kaplan-Meier survival curve of mice leukemia model (n=5 per group). *P < 0.05; **P < 0.01.

A total of 1×10^6^ C1498 cells transfected with NLRP3-GFP or Ctrl-GFP were intravenously injected into female WT C57BL/6J mice to establish AML mice. The AML mice were sacrificed after 21 days, and the expressions of NLRP3 related molecules were determined. Our results showed that the mRNA level of NLRP3, IL-1β or NF-κB was significantly elevated in BM of NLRP3-GFP AML mice compared with Ctrl-GFP AML mice (**Supplementary Figure 2F**). Moreover, the NLRP3-GFP AML mice presented with more severe hepatomegaly and splenomegaly in comparison to Ctrl-GFP mice (**Figure 2A**). The liver and spleen showed higher weight in NLRP3-GFP AML mice (**Figure 2B**). Leukemia cells in bone marrow or liver were examined by microscopy, and we observed that liver and bone marrow sections showed more leukemia cells infiltration in NLRP3-GFP AML mice compared with control mice (**Figure 2C**). Single-cell suspensions from spleen, liver or bone marrow were determined using flow cytometry analysis, and the results showed that the percentages of GFP+ leukemia cells in these organs from NLRP3-GFP AML mice were significantly higher than those from Ctrl-GFP AML mice (**Figure 2D**, **Supplementary Figure 2G**). Furthermore, NLRP3 up-regulation in C1498 led to a statistically shorter survival than Ctrl-GFP AML mice [median 22 (range 21-24) days vs median 27 (range 25-28) days (P=0.0188)] (**Figure 2E**). These results suggested that NLRP3 up-regulation in leukemia cells induces an expansion of leukemia cells in the BM, liver and spleen, and causes the poorer survival.

### Inactivation of NLRP3 Inflammasome by Caspase-1 or NF-κB Inhibitor Suppresses AML Leukemia Cells *In Vitro*

**Figure 3:**
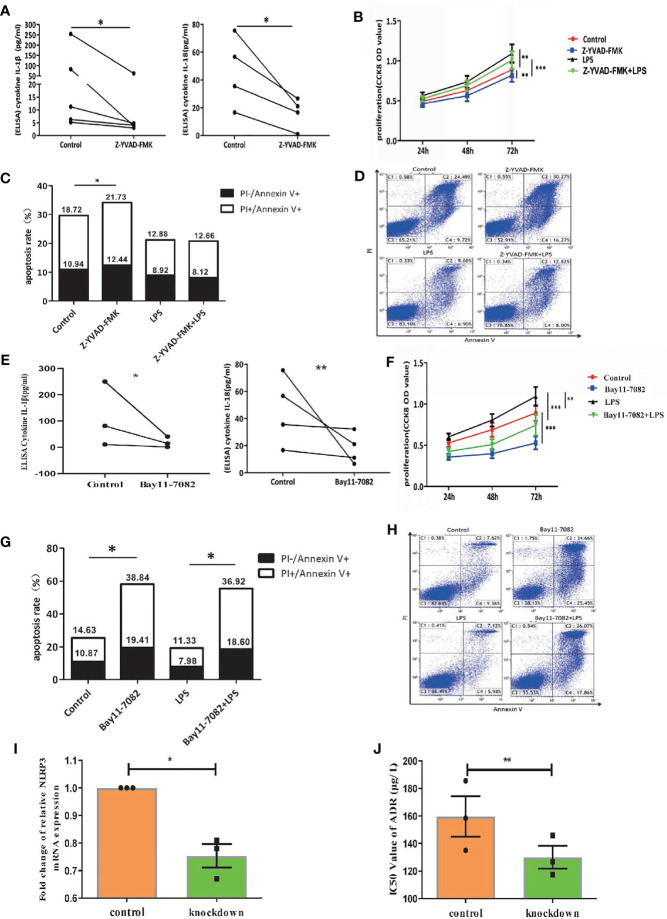
Inactivation of NLRP3 inflammasome by caspase-1 or NF-κB inhibitor suppresses AML leukemia cells *in vitro*. **(A)** The concentration of IL-1β (n=5) and IL-18 (n=4) in supernatants of cultured leukemia cells following Z-YVAD-FMK treatment. **(B)** Cell proliferation was analyzed by CCK8 assay in primary leukemia cells 24, 48 and 72 hours after treatment with LPS or/and Z-YVAD-FMK (n=8). **(C)** The apoptosis rate of leukemia cells in primary leukemia cells 48 hours after treatment with LPS or/and Z-YVAD-FMK (n=5). **(D)** Representative scatter plots of apoptosis. **(E)** ELISA results of secreted IL-1β (n=3) and IL-18 (n=4) by AML primary leukemia cells after treatment with Bay11-7082. **(F)** Cell proliferation was analyzed by CCK8 assay in primary leukemia cells 24, 48 and 72 hours after treatment with LPS or/and Bay11-7082 (n=7). **(G)** The apoptosis rate of leukemia cells in primary leukemia cells 48 hours after treatment with LPS or/and Bay11-7082 (n=3). **(H)** Representative scatter plots of apoptosis. **(I)** The results of NLRP3 mRNA expression in THP1 cells after being transfected with lentivirus (n=3). **(J)** The IC50 values of ADR for THP1 cells after downregulating NLRP3 expression (n=3). *P < 0.05; **P < 0.01; ***P < 0.001.

As caspase-1 is pivotal for the activation of NLRP3 inflammasome (7), we treated primary AML leukemia cells with caspase-1 inhibitor Z-YVAD-FMK. As expected, our results showed that Z-YVAD-FMK decreased the secretion of IL-1β and IL-18 into culture medium of leukemia cells (**Figure 3A**). CCK8 analysis showed Z-YVAD-FMK suppressed the proliferation of AML leukemia cells or LPS-activated AML cells (**Figure 3B**), and apoptosis analysis by flow cytometry showed Z-YVAD-FMK increased apoptosis rate of leukemia cells (**Figures 3C, D**). Our results confirmed that inhibiting caspase-1 in AML cells suppressed the pro-survival effect of NLRP3 inflammasome activation.

In addition, NF-κB signaling pathway is crucial for the NLRP3 inflammasome activation (7). We applied AML leukemia cells with NF-κB inhibitor Bay11-7082, and found that Bay11-7082 significantly decreased the protein levels of LPS+ATP induced pNF-κB, pro-caspase-1, pro-IL-1β, cleaved caspase-1 and cleaved IL-1β (**Figure 1C**, right column 1 and 3, **Supplementary Figure 1F**). Moreover, ELISA results showed that Bay11-7082 inhibited the secretion of IL-1β and IL-18 from AML primary leukemia cells (**Figure 3E**). CCK8 assay showed that Bay11-7082 inhibited the proliferation of AML leukemia cells or LPS-activated AML cells (**Figure 3F**). In addition, we found that the apoptosis in primary leukemia cells with or without LPS activation was apparently enhanced in the presence of Bay11-7082 (**Figures 3G, H**). Furthermore, to explore the endogenous deficiency of NLRP3 in AML, we knocked down NLRP3 in leukemia cells by using siRNA transfection (**Figure 3I**). The result showed that the deficiency of NLRP3 increased the drug sensitivity with the decreasing IC50 of ADR (from 160 to 127 µg/L) (**Figure 3J**). These observations collectively indicated that inactivation of NLRP3 inflammasome suppresses the progression in leukemia cells.

### Knockout of NLRP3 Attenuates Leukemia Burden in AML Mice

**Figure 4:**
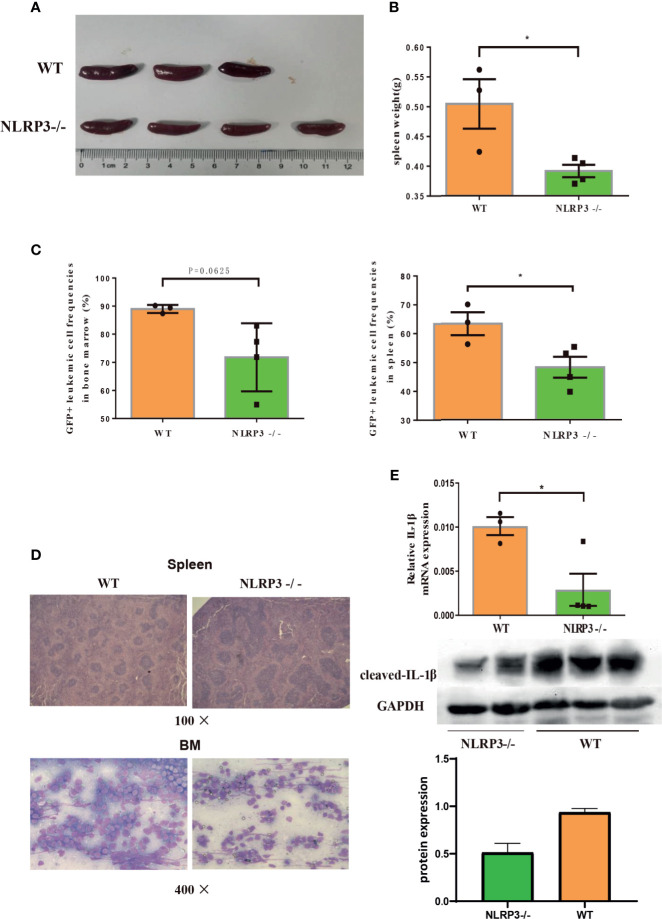
Knockout of NLRP3 attenuates leukemia burden in AML mice. **(A)** Representative photographs of spleen from WT mice (n=3) and NLRP3-/- mice (n=4). **(B)** The weight of spleens of WT mice (n=3) and NLRP3-/- mice (n=4). **(C)** The leukemia cells (GFP+ cells) in spleen and bone marrow from WT mice (n=3) and NLRP3-/- mice (n=4). **(D)** Hematoxylin and eosin-stained histopathology sections of a representative spleen and bone marrow from WT and NLRP3-/- mice (100× or400×). **(E)** The mRNA and protein expressions of IL-1β by qRT-PCR and western- blot in bone marrow of WT and NLRP3 -/- mice. *P < 0.05.

Furthermore, we continued to investigate the effect of NLRP3 in AML murine model. The *NLRP3-/-* AML mice or control AML mice were successful constructed and certificated (**Supplementary Figure 2H**). Then, we designed two groups of C57BL/6J AML mice, one for injection with *NLRP3-/-* AML leukemia cells and one for injection control AML leukemia cells. We found that *NLRP3-/-* AML mice exhibited a milder splenomegaly and a lighter spleen weight compared to control AML mice (**Figures 4A, B**). Moreover, the results showed that the percentage of GFP+ cells in the spleen and bone marrow of *NLRP3-/-* AML mice was lower than that in the control AML mice (**Figure 4C**). As shown by microscopy, leukemia cells infiltration in spleen and bone marrow showed alleviated tendency in *NLRP3^-/-^* mice compared with those in control AML mice (**Figure 4D**). Furthermore, our results found that *NLRP3^-/-^* murine bone marrow cells showed less IL-1β expression at mRNA and protein levels (**Figure 4E**).

### The Leukemia-Promoting Effect Induced by NLRP3 Activation Acts Through IL-1β but Mot IL-18

**Figure 5:**
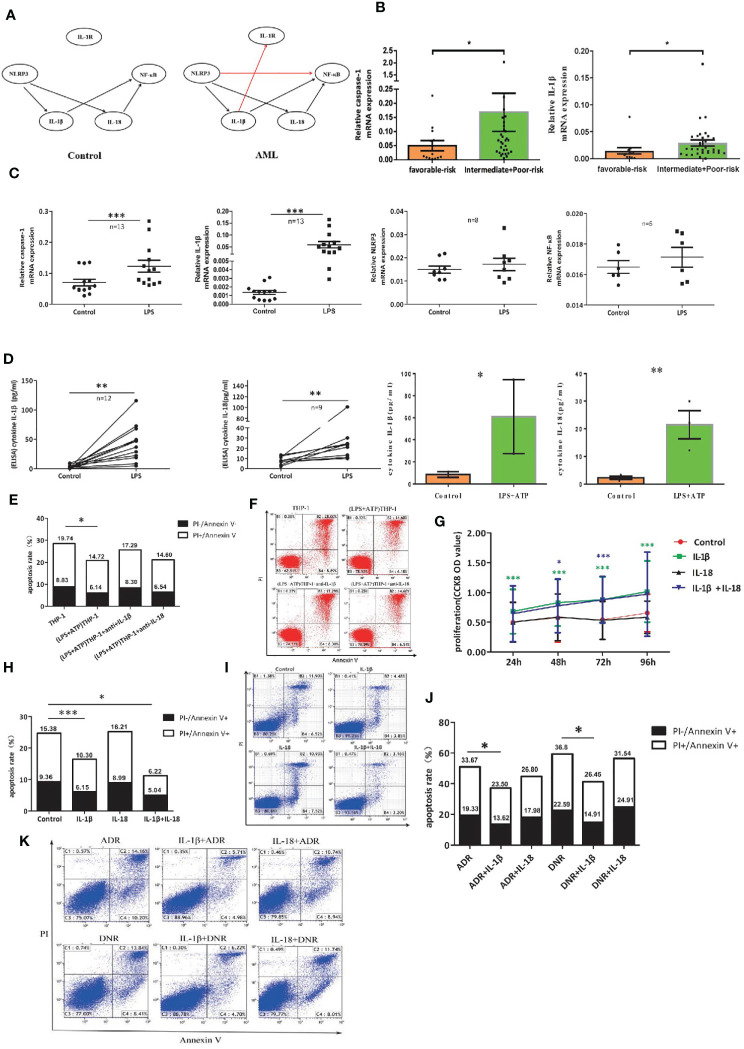
The leukemia-promoting effect induced by NLRP3 activation acts through IL-1β but not IL-18. **(A)** Bayesian network model diagram was designed according to qRT-PCR results of NLRP3 inflammasome in controls and ND AML patients. **(B)** The mRNA expressions of NLRP3 inflammasome components caspase-1 and IL-1β in favorable (n=13) and intermediate/poor-risk (n=32) groups. **(C)** The mRNA expressions of NLRP3 inflammasome components caspase-1, IL-1β, NLRP3 and NF-κB in THP-1 cells were compared before and after LPS stimulation. **(D)** The concentrations of IL-1β and IL-18 in supernatants of cultured THP-1 cells following LPS or LPS+ATP treatment. **(E)** The apoptosis rate of primary leukemia cells after being co-cultured with LPS-activated THP1 cells with or without adding anti-IL-1β or anti-IL-18 antibody (n=6). **(F)** Representative scatter plots of apoptosis. **(G)** CCK8 assay was applied to analyze the proliferation of primary leukemia cells after adding IL-1β or/and IL-18 for 24, 48, 72 and 96 hours (n=21). **(H)** Flow cytometry analysis of Annexin V-FITC/PI-staining method was performed to analyze apoptosis of primary leukemia cells after adding IL-1β or/and IL-18 for 48 hours (n=24). **(I)** Results were plotted as the percentage of cells in each quadrant. **(J)** Cell apoptosis was analyzed by flow cytometry 48 hours after adding ADR (200 μg/L) or DNR (20 μg/L) with or without IL-1β or IL-18 (n=8). **(K)** Representative scatter plots of flow cytometry. *P < 0.05; **P < 0.01; ***P < 0.001.

Bayesian network analysis is usually used to analyze the relationship among many molecules. To clarify the complicated correlation structure and potential signaling pathway among the genes in NLRP3 inflammasome, we used score-based hill-climbing greedy search algorithm based on mRNA expression data to learn the network structure in AML patients and controls respectively. By comparison with that for control group, the end of network for AML patients was pointed from IL-1β to IL-1R and from NLRP3 to NF-κB (**Figure 5A**). Furthermore, significantly higher expression of caspase-1 or IL-1β was found in AML intermediate/poor risk classification compared with favorable risk group (**Figure 5B**). All these findings illustrate the evidence that highly activated NLRP3 inflammasome in AML bone marrow leukemia cells correlates with poor prognosis and may act through IL-1β pathway in AML.

To further investigate the role of IL-1β in the leukemia-promoting effect by NLRP3 inflammasome activation, we first activated NLRP3 inflammasome of THP-1 cells with LPS or LPS+ATP and found that the mRNA expression of caspase-1 or IL-1β and the secreted IL-1β and IL-18 were significantly increased after NLRP3 activation (**Figures 5C, D**). Then, we co-cultured NLRP3-activated THP-1 cells with primary leukemia cells using Transwell chamber. Our results showed that the culture medium of NLRP3-activated THP-1 cells inhibited primary leukemia cells apoptosis, and the inhibitory effect had partially reversed after neutralization with anti-IL-1β, while anti-IL-18 had no this effect (**Figures 5E, F**). Furthermore, human recombinant IL-1β and/or IL-18 were added into the culture medium of primary AML leukemia cells. CCK8 results showed that IL-1β or IL-1β+IL-18 significantly stimulated the proliferation and inhibited the apoptosis of leukemia cells, but IL-18 alone had no obvious effects (**Figures 5G-I**). As for the influence on the efficacy of chemotherapy, our results showed that IL-1β significantly attenuated the antitumor effect of ADR or DNR. In contrast, IL-18 had little effect on antitumor effect of chemotherapeutic drugs (**Figures 5J, K**). These findings verified that IL-1β but not IL-18 is critical for the resistance to the cytotoxicity of ADR and DNR in AML leukemia cells.

### Down-Regulation of Endogenous IL-1β Suppresses the Growth of Leukemia Cells

**Figure 6:**
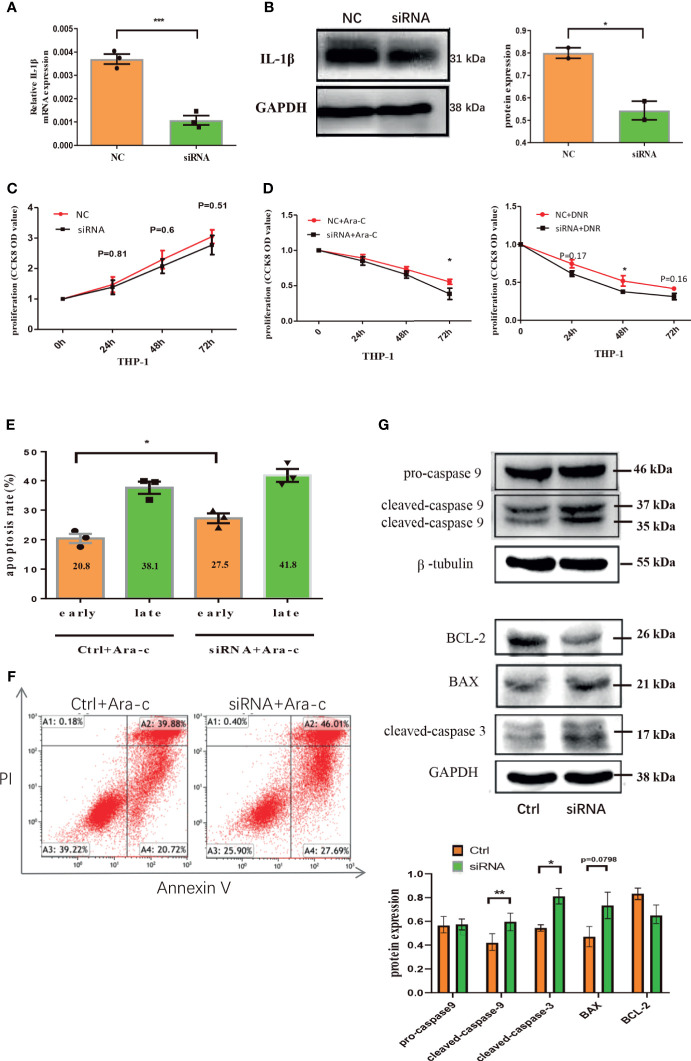
Down-regulation of endogenous IL-1β suppresses the growth of leukemia cells. The mRNA expression of IL-1β was determined by qRT-PCR in THP1 cells after IL-1β siRNA transfection (n=3) **(A)** and the protein level of IL-1β by western blot in THP1 cells after IL-1β siRNA transfection was also shown (n=3) **(B)**. The proliferation results by CCK8 assay for leukemia cells 24, 48, 72 hours after knocking down IL-1β by siRNA transfection (n=3) **(C)** and treatment with drugs (n=3) **(D)**. The apoptosis of THP1 cells was quantified 48 hours after IL-1β knockdown by siRNA (n=3) **(E)** and the representative scatter plots of apoptosis **(F)**. **(G)** The western blot results of pro-caspase-9, cleaved caspase-9, and β-tubulin is a loading control. Representative western blot bands of Bcl-2, BAX, cleaved caspase-3, and GAPDH is a loading control (n=3). *P < 0.05; **P < 0.01; ***P < 0.001.

To further clarify the role of endogenous IL-1β in AML leukemia cells, IL-1β siRNA was transfected into THP-1 leukemia cells, which significantly decreased the mRNA or protein level of IL-1β (**Figures 6A, B**). CCK8 assay showed that the proliferation was inhibited in IL-1β siRNA transfected leukemia cells compared with controls after treatment with Ara-C or DNR, though IL-1β knockdown alone has no statistical effect on THP-1 cells (**Figures 6C, D**). Moreover, IL-1β knockdown significantly increased the apoptosis rate of leukemia cells after treatment of chemotherapy (**Figures 6E, F**). Mechanistically, we determined the expression of signaling molecules associated with proliferation and apoptosis by western blot. Our results showed that the apoptosis proteins, cleaved caspase 3, cleaved caspase 9 and Bax, were aberrantly over-expressed after knocking down IL-1β in leukemia cells by siRNA, while Bcl-2 onco-protein was down-regulated (**Figure 6G**). These findings suggested that endogenous knockdown of IL-1β suppresses leukemia cells.

## Discussion

Chronic inflammatory responses were reported to be associated with many cancers (13–15). The NLRP3 inflammasome is one of the best-characterized inflammasomes that is involved in chronic inflammatory responses (4, 5). NLRP3 inflammasome has also been found related to tumor pathogenesis, but its role in cancer is versatile and sometimes controversial (9, 12–14). The biological relationship between inflammasome and cancer may provide a novel approach for anticancer therapies. AML is one of the most malignant diseases threatening the health worldwide and our previous study found that the genetic polymorphisms of IL-18 rs1946518 and IL-1β rs16944 are associated with prognosis and survival of AML patients (31). However, the exact functional role or production mechanism of NLRP3 inflammasome in the development and treatment of AML remains to be fully identified. In this study, we uncovered a previously unknown and unexpected role of NLRP3 inflammasome in AML.

Overexpression of inflammasome components has been reported in various types of cancer, such as head and neck squamous cell carcinoma (23, 33) lung cancer (34), gastric cancer (35) and breast cancer (33). In the present study, we documented that primary AML leukemia cells over-expressed NLRP3 inflammasome associated molecules at the mRNA and protein levels which were correlated with poor risk of AML patients. Further, the secreted IL-1β level in BM plasma was also found up-regulated. All these findings suggested that the higher expression and activity of NLRP3 inflammasome in bone marrow leukemia cells may play a vital role in AML.

As shown in the scope of solid tumors, the effects of NLRP3 inflammasome are cell- and tissue-specific (13–15). Though the inflammasome activation and IL-18 signaling pathways are largely beneficial in colitis-associated colorectal cancer (32, 36), it has been widely accepted that inflammasome can promote the development of many malignant tumors, including head and neck squamous cell carcinoma, fibrosarcoma, melanoma, gastric carcinoma and lung metastasis (9, 10, 12–14, 37). Recent evidences suggested that activation of NLRP3 inflammasome by mycoplasma hyorhinis promotes gastric cancer migration and invasion (38), and this gastric tumor-promoting effect by NLRP3 may be due to the activating CCND1 transcription (35). Another study indicated that NLRP3 expression in infiltrating macrophages was significantly associated with survival and metastasis in human breast tumor *via* S1PR1 signaling (33). As NLRP3 inflammasome agonists are structurally heterogeneous, most researchers activated NLRP3 inflammasome pathway *via* classical combination of LPS+ATP (4, 7). It was reported that LPS+ATP induced activation of NLRP3 inflammasome in A549 lung cancer cells, and NLRP3 inflammasome activation enhanced the proliferation and migration of A549 cells by secreting IL-1β and IL-18 (17). In human monocytes, LPS alone can activate an alternative NLRP3 inflammasome pathway and stimulate IL-1β secretion (8). In this study, in primary AML leukemia cells, LPS or LPS+ATP induced NLRP3 inflammasome activation and the activation of NLRP3 inflammasome promoted proliferation, inhibited apoptosis and increased drug-resistance of primary leukemia cells. Additionally, we up-regulated the expression of NLRP3 in the murine leukemia cell line C1498 by lentivirus. At the same time, we also transplanted the bone marrow of the *NLRP3^-/-^* mouse into normal mice. We found that NLRP3 inflammasome caused a worse outcome and more leukemia infiltration. These results indicated that NLRP3 activation may play a carcinogenetic role in AML.

In this study, we further explored the effect of inhibition of NLRP3 inflammasome activity in AML. Many studies suggested that inhibition or inactivation of NLRP3 inflammasome plays an antitumor role in many cancers. It was reported that the inhibition of NLRP3 inflammasome by MCC950 delayed the progression of tumor growth in mice with head and neck squamous cell carcinoma (23). And a recent study demonstrated that inhibiting NLRP3 inflammasome and limiting IL-1β secretion were the main mechanisms of miR-22-induced decreased gastric cancer cells proliferation (35). As caspase-1 was the major downstream molecule of NLRP3 inflammasome, its inhibitor Z-YVAD-FMK attenuated LPS+ATP-induced A549 lung cancer cells proliferation (17). Our results showed that caspase-1 inhibitor Z-YVAD-FMK down-regulated IL-1β and IL-18 secretion, suppressed proliferation and enhanced apoptosis of primary leukemia cells. Additionally, NFκB is involved in NLRP3 inflammasome activation and its pro-tumorigenic role has been widely described in cancer (14). The NF-κB inhibitor Bay 11-7082 is a potent and selective inhibitor of NLRP3 inflammasome activation independent of their inhibitory effect on the NF-κB activity (39). Here, we demonstrated that NF-κB inhibitor Bay11-7082 decreased the secretion of IL-1β and IL-18 from leukemia cells and down-regulated the protein expression of pNF-κB, pro-caspase-1, pro-IL-1β, active caspase-1 and IL-1β. And inactivation of NLRP3 inflammasome by inhibiting NF-κB can also inhibit the proliferation and induce the apoptosis of AML cells.

NLRP3 inflammasome pathway is a complex signal network consisting of many interactive molecules. We used bioinformatics methods to clarify the relationship among these molecules determined in our cohort of AML patients. Bayesian networks consist of nodes and arcs that represented variables of relationships between them (40, 41). Given its advantage of visual presentation and network structures that were more appropriate to describe interactions between variables, Bayesian networks were used in medical (42), biological (43), and social research to study the conditional dependencies between random variables. Therefore, we used score-based hill-climbing greedy search algorithm to learn the Bayesian network of NLRP3 inflammasome. Compared with the controls, the end of network for AML patients was pointed from IL-1β to IL-1R and from NLRP3 to NF-κB, which indicates that NLRP3 inflammasome may act through IL-1β or NF-κB in AML leukemia cells.

IL-1 affects the process of carcinogenesis, tumor growth and invasiveness at tumor sites in many kinds of cancers (44). As the main effectors of NLRP3 inflammasome, IL-1β and IL-18 belong to the IL-1 superfamily and have the potential to promote an immune-suppressive tumor microenvironment. IL-1β is one of the critical pro-inflammatory cytokines involved in tumor pathogenesis (17–21). IL-1β has been reported to contribute to the development and progression of melanoma (45). It was also demonstrated that IL-1β enhances migration and invasion in oral cancer (46) and gastric cancer (47) by down-regulating E-cadherin and up-regulating Snail. Moreover, IL-18 enhances angiogenesis and promotes tumor cell proliferation and migration in gastric cancer (48–50). Our previous study found IL-18 mRNA expression and plasma IL-18 level were increased in lymphoma patients. Moreover, IL-18 promoted proliferation, inhibited apoptosis and reduced the anti-tumor effect of dexamethasone for lymphoma cells (24). In this study, we found IL-1β accelerated proliferation and inhibited apoptosis of AML cells, and significantly attenuated the antitumor effect of ADR and DNR, but IL-18 showed little effect. It is speculated that NLRP3 inflammasome promotes tumorigenesis in AML mainly *via* IL-1β pathway.

In conclusion, our results suggested that a hallmark of AML is activation of the NLRP3 inflammasome and NLRP3 inflammasome functions as an oncogenic factor through IL-1β pathway in AML. Regulating NLRP3 inflammasome activity especially targeting IL-1β may provide a novel approach for AML therapy.

## Data Availability Statement

The raw data supporting the conclusions of this article will be made available by the authors, without undue reservation.

## Ethics Statement

The animal study was reviewed and approved by Qilu Hospital of Shandong University.

## Author Contributions

DM and CJ designed and funded the research. CZho, RW and MH performed the research and contributed equally to this study. CZha and FH assisted the research. XY and XH analyzed the data. CZho wrote the paper. MX edited the paper. GL and TS helped in funding this research. All authors contributed to the article and approved the submitted version.

## Funding

This work was supported by grants from National Natural Science Foundation of China (No.81873439, 91642110, 81800157, 81873425), Shandong Key Research and Development Program (2017GSF218050), the Fundamental Research Funds of Shandong University (2018JC018), and grants from Taishan Scholars Program (tspd20210321, tsqn201812132).

## Conflict of Interest

The authors declare that the research was conducted in the absence of any commercial or financial relationships that could be construed as a potential conflict of interest.
