# Corrigendum: NLRP3 Inflammasome Promotes the Progression of Acute Myeloid Leukemia *via* IL-1β Pathway

**DOI:** 10.3389/fimmu.2021.808492

**Published:** 2021-11-16

**Authors:** Chaoqin Zhong, Ruiqing Wang, Mingqiang Hua, Chen Zhang, Fengjiao Han, Miao Xu, Xinyu Yang, Guosheng Li, Xiang Hu, Tao Sun, Chunyan Ji, Daoxin Ma

**Affiliations:** ^1^Department of Hematology, Qilu Hospital of Shandong University, Jinan, China; ^2^Department of Hematology, Shandong Yantai Mountain Hospital, Yantai, China; ^3^Department of Emergency, Qilu Hospital of Shandong University, Jinan, China

**Keywords:** inflammasome, NLRP3, IL-1β, acute myeloid leukemia, progression

An author name was incorrectly spelled as **Chaoqing Zhong**. The correct spelling is **Chaoqin Zhong**.

The authors apologize for this error and state that this does not change the scientific conclusions of the article in any way. The original article has been updated.

## Publisher’s Note

All claims expressed in this article are solely those of the authors and do not necessarily represent those of their affiliated organizations, or those of the publisher, the editors and the reviewers. Any product that may be evaluated in this article, or claim that may be made by its manufacturer, is not guaranteed or endorsed by the publisher.

