# Supplementary material for: NLRP3 Inflammasome Promotes the Progression of Acute Myeloid Leukemia via IL-1β Pathway
Source: Front Immunol. 2021 Jun 15;12:661939. doi: 10.3389/fimmu.2021.661939 (PMC8239362; doi:10.3389/fimmu.2021.661939)
Supplement: Supplementary file 1 [file Image_1.pdf]

Supplementary Figure 1

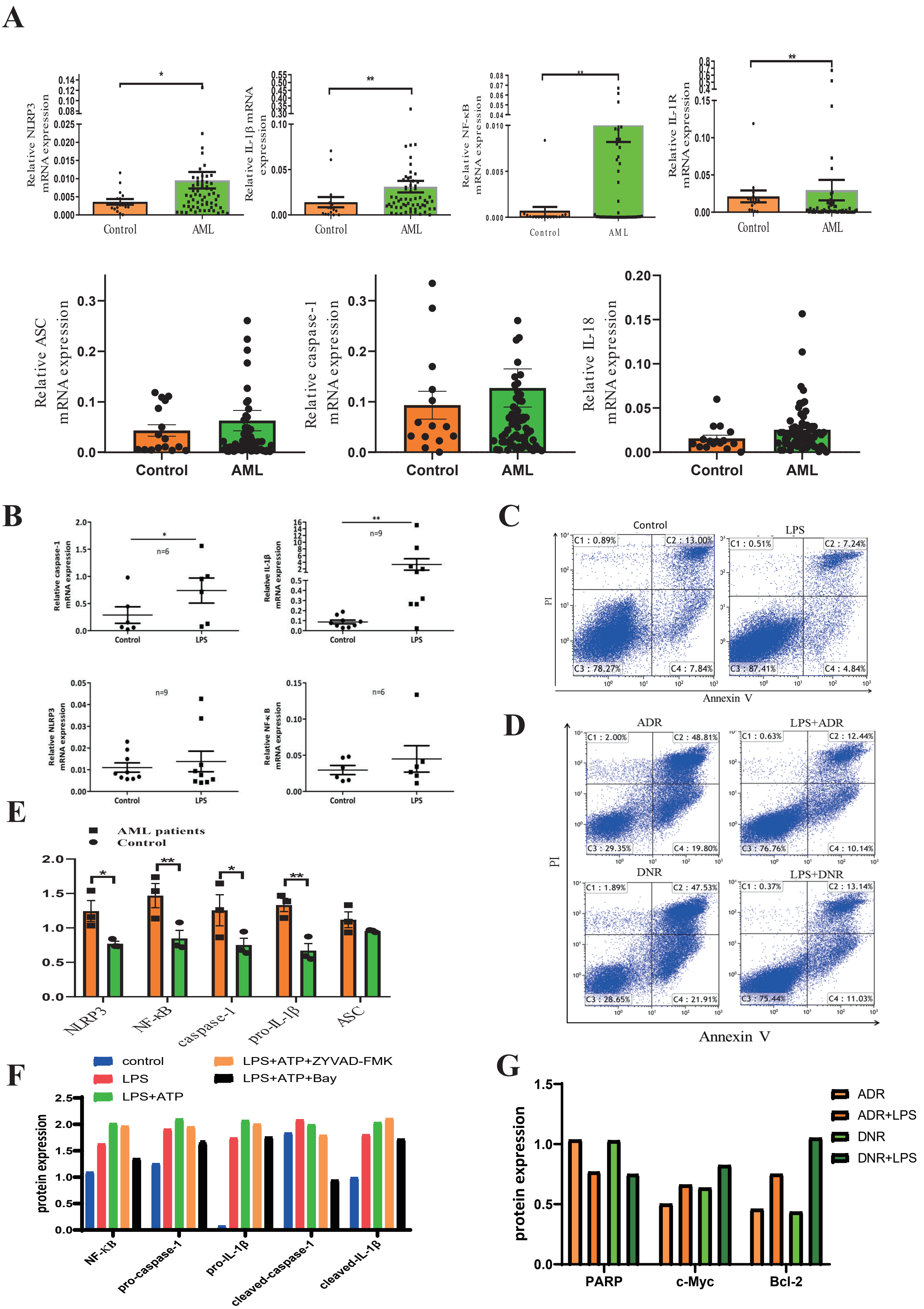

Supplementary Figure 1. The additional results in vitro experiment. (A) The mRNA expression levels of NLRP3 inflammasome components NLRP3, IL-1β, NF-κB, IL-1R, ASC, caspase-1 and IL-18 in BM-MNCs isolated from ND AML patients (n=63) and controls (n=16). (B) The mRNA expression levels of NLRP3, caspase-1, IL-1β and NF-κB in primary leukemia cells were compared between LPS stimulation and controls. (C) The representative FACS graphs of apoptotic cells of leukemia cells were shown after being cultured with LPS for 48 hours. (D)The representative FACS graphs of apoptotic cells of leukemia cells for Figure 1I. (E -G)Densitometry analysis for the results of western blots for Figure 1A, C an.d L.

Supplementary Figure 2

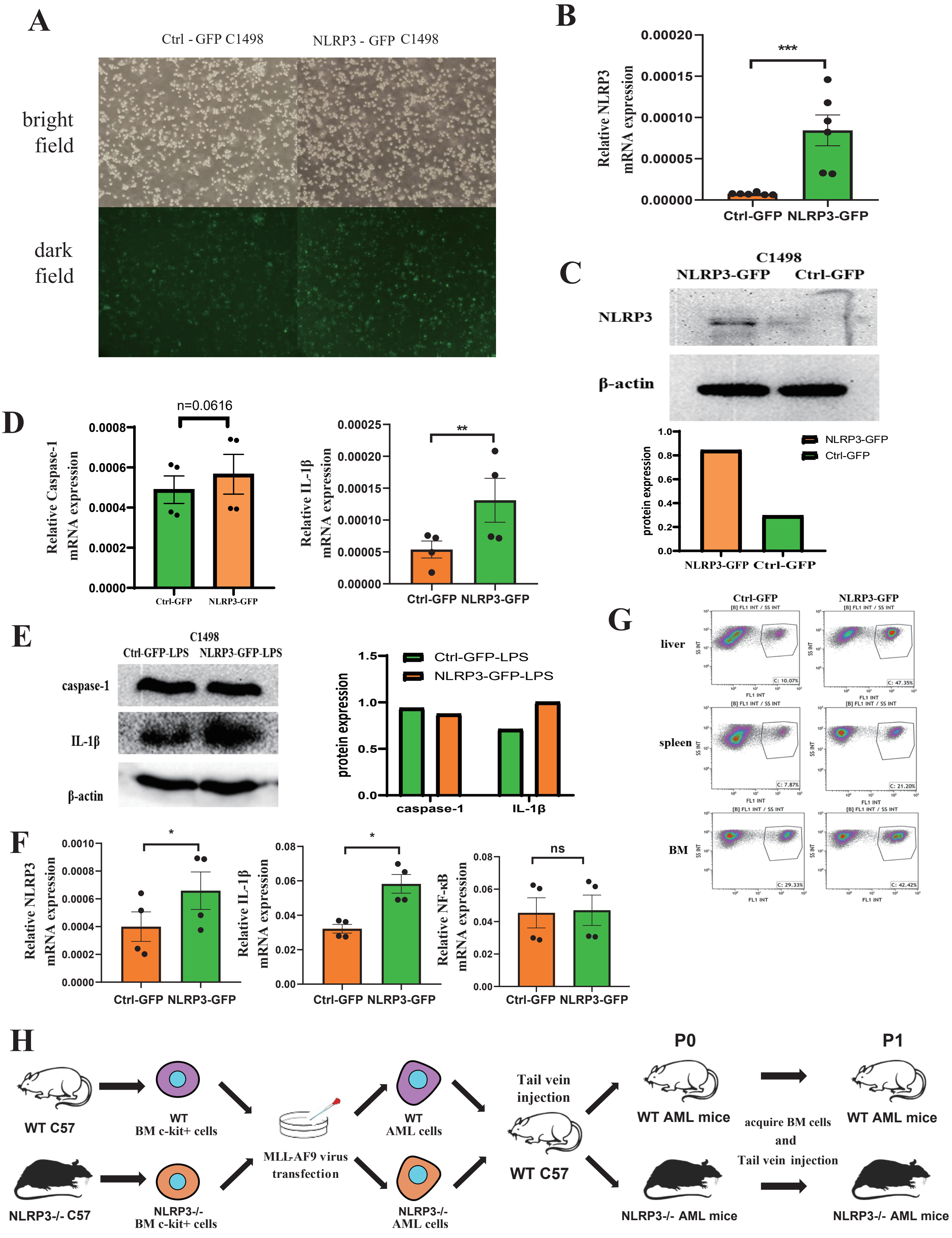

Supplementary Figure 2. The additional results in vivo experiment.

(A) C1498 cells were infected with NLRP3-GFP or Ctrl-GFP lentivirus for 48 hours, the efficiency of infection was detected with fluorescence microscope (original magnification×100). (B) The qRT-PCR results of NLRP3 for C1498 cells after transfection with NLRP3 lentivirus (n=6). (C) The Western blot results of NLRP3 protein for C1498 cells after transfection with NLRP3 lentivirus. (D) The qRT-PCR results of caspase-1 and IL-1β for NLRP3-GFP transfected C1498 cells after LPS activation (n=4). (E) The Western blot results of caspase-1 and IL-1β for NLRP3-GFP transfected C1498 cells after LPS activation. (F) The mRNA expression levels of NLRP3, IL-1β and NF-κB in BM-MNCs isolated from NLRP3-GFP AML mice (n=4) compared with Ctrl-GFP AML mice(n=4). (G) The representative FACS graphs of spleen, liver and bone marrow swelling from normal, Ctrl-GFP and NLRP3-GFP mice. (H) Mouse model establishment process.
